# Optimizing [^99m^Tc]Tc-Tamsulosin for Enhanced Tumor Detection: A Comprehensive Approach from Preparation to In Vivo Evaluation

**DOI:** 10.1007/s13139-025-00983-5

**Published:** 2026-02-18

**Authors:** M. H. Sanad, Safaa B. Challan, A. Massoud

**Affiliations:** 1 Labeled Compounds Department, Hot Laboratories and Waste Management Center, Egyptian Atomic Energy Authority, P.O. Box 13759, Cairo, Egypt; 2 Cyclotron Facility, Nuclear Research Center, Egyptian Atomic Energy Authority, P.O. Box 13759, Cairo, Egypt; 3 Nuclear Chemistry Department, Hot Laboratories and Waste Management Center, Egyptian Atomic Energy Authority, P.O. Box 13759, Cairo, Egypt

**Keywords:** Tamsulosin hydrochloride, *T*echnetium-99m [^99m^Tc], Radiolabeling optimization, Tumor imaging, In-vivo evaluation

## Abstract

**Purpose:**

To investigate the potential of Tamsulosin hydrochloride, a selective α1-adrenoceptor antagonist, as a tumor-targeting ligand when labeled with Technetium-99 m [⁹⁹ᵐTc] for use as a novel radiopharmaceutical. This study aims to develop and evaluate the [⁹⁹ᵐTc]Tc-Tamsulosin complex for enhanced tumor imaging and improved diagnostic accuracy in oncology.

**Methods:**

The radiolabeling process was optimized using 0.6 mg of tamsulosin, 75 µg of SnCl_2_.2H_2_O and 200 µL of pH 4 buffer with 0.1 ml (350 MBq) Na[^99m^TcO_4_]^−^, with the mixture incubated at room temperature (25°C) for 30 min. Radiochemical yield and in vitro stability were assessed via TLC, and purification was performed through electrophoresis and high-performance liquid chromatography (HPLC), followed by in vivo evaluation studies in tumor-bearing mice versus normal mice.

**Results:**

The [⁹⁹ᵐTc]Tc-Tamsulosin complex achieved a radiochemical purity of 97.5 ± 0.12% and maintained in vitro stability in serum and PBS above 87 ± 0.22% after 24 h. In vivo evaluation revealed peak tumor uptake (T) of 5.2 ± 0.02% ID/g and normal muscle (NT) uptake of 0.98 ± 0.01% ID/g at 2 h post-injection, with a target-to-non-target ratio of 5.3. Renal clearance was prominent, with kidney uptake of 16 ± 0.24% ID/g and urinary excretion reaching 25 ± 1.37% ID/g at 3 h.

**Conclusion:**

The [⁹⁹ᵐTc]Tc-Tamsulosin complex demonstrates high radiolabeling efficiency, stability, and selective tumor accumulation, supporting its potential as a promising radiopharmaceutical for accurate tumor imaging. These findings highlight its value in advancing nuclear medicine oncology diagnosis.

## Highlights

- Objectives:
  - To investigate the radiolabeling of Tamsulosin with Technetium-99m (⁹⁹ᵐTc) as a potential radiopharmaceutical for enhanced tumor detection.
- Radiolabeling Conditions:
  - ○ Tamsulosin ligand: 0.6 mg
  - ○ Reducing agent: 75 µg of stannous chloride (SnCl₂)
  - ○ Buffer: 200 µl at pH 4
  - ○ Temperature: Room temperature (25 °C)
  - ○ Incubation time: 30 minutes
- Radiochemical Yield and Purity:
  - ○ Radiolabeled complex: [^99m^Tc]Tc-Tamsulosin
  - ○ Purification methods: Electrophoresis and high-performance liquid chromatography (HPLC)
  - ○ Maximum labeling yield: 97.5 ± 0.12%
  - ○ Stability: Maintained for over 4 hours post-labeling
- In Vivo Biodistribution:
  - ○ Significant tumor-specific accumulation observed
  - ○ Tumor-to non-target uptake ratio: 5.3 at 2 hours post-injection
- Conclusion:
  - The study demonstrates that [^99m^Tc]Tc-Tamsulosin exhibits high radiochemical purity, in vivo stability, and selective tumor uptake, underscoring its potential as a promising diagnostic agent in oncological nuclear imaging.

## Introduction

Cancer is a heterogeneous disease characterized by the uncontrolled proliferation of abnormal cells, which can invade surrounding tissues and disrupt organ function [1]. It remains a leading global health challenge, contributing to significant morbidity and mortality across populations. According to the World Health Organization (WHO), nearly 10 million cancer-related deaths were reported in 2020, underscoring the urgent need for innovative therapeutic strategies [2]. The complexity of the disease, driven by its heterogeneous nature, dynamic progression, and multifactorial etiology, necessitates continuous advancement in drug development and clinical intervention. In this context, the exploration of novel anticancer agents and experimental models has become essential to enhancing therapeutic efficacy and facilitating patient recovery [3].

Among the experimental platforms used to study tumor biology and therapeutic effectiveness, Ehrlich ascites carcinoma (EAC) provides a versatile and clinically relevant model. Derived from a spontaneous murine mammary adenocarcinoma, EAC can be grown in ascitic or solid forms, allowing simulation of both cavity-based and localized solid tumors [4]. Its rapid growth, immunogenic characteristics, and response to chemotherapeutic agents make it a valuable tool for preclinical testing of radiopharmaceuticals and apoptosis-inducing compounds. Depending on how it is administered intraperitoneally, subcutaneously, or intramuscularly, EAC can mimic different tumor microenvironments, facilitating targeted studies of drug delivery, tumor-host interactions, and biological responses [5].

In its solid form, EAC tumors display characteristic features of the tumor microenvironment (TME), including fibrin-rich stroma formation, angiogenesis, and immune cell infiltration. Plasma-derived fibrinogen is quickly converted into fibrin by tumor-associated procoagulants, creating a gel matrix that organizes malignant cells into nests and supports stromal development [6]. This scaffold helps recruit fibroblasts, macrophages, and endothelial cells, aiding in vascular remodeling and tumor growth. The evolving TME is marked by cytokine signaling, extracellular matrix remodeling, and infiltration of immune suppressor cells. These features make EAC a reliable and anatomically specific model for studying radiopharmaceutical targeting, therapeutic effectiveness, and mechanisms of tumor biology [7].

Adrenoceptors, also known as adrenergic receptors, are G-protein-coupled receptors that are classified into alpha (α1 and α2) and beta (β1, β2, and β3) subtypes. Each subtype demonstrates distinct distributions and functions in physiological processes such as vasoconstriction, metabolism, and neural signaling in mice [8]. These receptors are distributed throughout the mouse body, including the central nervous system (CNS), cardiovascular system, gastrointestinal tract, and skeletal muscles [9]. In the thigh muscles (hindlimb skeletal muscles such as the soleus, plantaris, and gastrocnemius), β2-adrenoceptors are the predominant subtype on muscle fibers, potentially facilitating protein metabolism and hypertrophy. Conversely, α1-adrenoceptors are located on the sarcolemma of specific fiber types. In mouse skeletal muscle, α1-adrenoceptors are implicated in modulating processes such as glucose uptake, blood flow, and possibly contractile properties, although their density is relatively low [10].

**Fig. 1:**
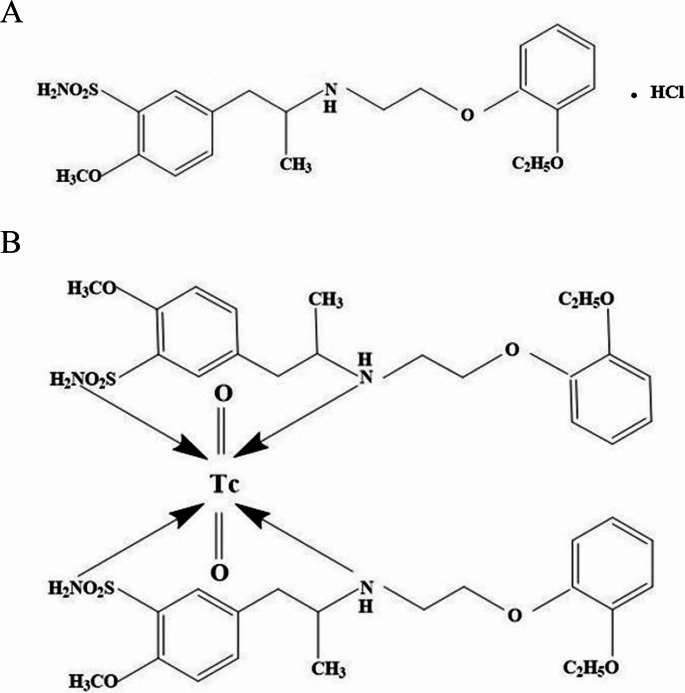
(**a**) Tamsulosin hydrochloride structure. (**b**) Proposed coordination structure of tamsulosin with technecium-99 m

Tamsulosin hydrochloride (Tamsulin), a selective α1-adrenoceptor antagonist, is widely employed in urological practice for the management of benign prostatic hyperplasia (BPH) [11]. It mitigates lower urinary tract symptoms by promoting the relaxation of smooth muscle in the prostate and bladder neck [12]. Additionally, tamsulosin has exhibited therapeutic versatility in addressing chronic prostatitis, pelvic pain syndrome, kidney stone passage, ureteral stent discomfort, urethral obstruction, and overactive bladder. The chemical designation for tamsulosin hydrochloride is 5-(2R)−2-[[2-(2-ethoxyphenoxy) ethyl] amino]propyl]−2-methoxybenzenesulfonamide hydrochloride, as illustrated in Fig. 1a. Tamsulosin hydrochloride is categorized as an aryl sulfonamide derivative [13]. Notably, sulfonamide groups have garnered significant interest in oncology due to their documented antitumor activities. Sulfonamide-based compounds exert anticancer effects through various mechanisms, with carbonic anhydrase (CA) inhibition being particularly noteworthy [14]. This inhibition disrupts cellular bicarbonate supply, thereby interfering with processes such as nucleotide synthesis and membrane lipid formation, which are essential for tumor growth [15].

Nuclear medicine has become integral to cancer diagnosis, with imaging techniques like SPECT and PET providing high-resolution, non-invasive visualization of tumors. Among the various radioisotopes used in molecular imaging, technetium-99m [^99m^Tc] is distinguished by its advantageous physical properties and versatility, with a half-life of 6 h and low-energy gamma emission of 140 keV. [^99m^Tc] is optimally suited for imaging applications, allowing sufficient time for diagnostic procedures while minimizing patient exposure to ionizing radiation [16]. Recent advancements in tumor imaging have introduced a range of technetium-99m labeled radiopharmaceuticals, including [^99m^Tc]oseltamivir, [^99m^Tc]dioxime, [^99m^Tc]ifosfamide, [^99m^Tc]amitrole, [^99m^Tc]mebeverine, [^99m^Tc]gemcitabine, [^99m^Tc]lutelin, [^99m^Tc]chlorambucil, [^9m^Tc]sunitinib, and [^99m^Tc]azathioprine [17–26]. These tracers demonstrate promising tumor-targeting capabilities, with selective binding to malignant cells that significantly enhances diagnostic precision and may inform therapeutic decision-making. Nevertheless, achieving a high degree of selectivity, particularly a target-to-non-target (T/NT) uptake ratio exceeding 1.5, remains a critical challenge in the development of radiopharmaceuticals with robust tumor specificity and clinical utility. The ability of these tracers to selectively bind to tumor cells represents a significant advancement in oncology, enabling more accurate diagnosis and potentially guiding therapeutic interventions.

The present study examines the radiolabeling of tamsulosin with technetium-99 m. A systematic analysis was conducted to investigate various parameters that influence the radiochemical yield, including the quantity of tamsulosin, the amount of reducing agent, pH, and incubation time. Additionally, the radiochemical yield and in vitro stability in serum and PBS were assessed using thin-layer chromatography (TLC). Purification was achieved through electrophoresis and high-performance liquid chromatography (HPLC), followed by in vivo evaluation studies in tumor-bearing mice compared to normal mice.

## Experimental

### Materials and Methods

Chemicals and reagents were obtained from Sigma-Aldrich Company in Cairo, Egypt. Tamsulosin hydrochloride (C_20_H_28_N_2_O_5_S.HCl), with a low molecular weight of 444.98 g/mol, was obtained from Marcy Pharmaceutical Co., Cairo, Egypt. Stannous chloride powder and pH buffer solutions ranging from 2 to 10 were purchased from Sigma (USA). Thin-layer chromatography (TLC) aluminum sheets (20 × 25 cm, SG-60 F254) from Merck (Germany) and paper chromatography from International Ltd. (UK). A paper electrophoresis (PE) device, featuring a 300 V power supply and chamber unit, was purchased from E.C. Apparatus Corporation (USA). Radiochemical purification was performed using an HPLC system with a Scalar Ratemeter (UK) equipped with a C_18_ reverse-phase column (25 cm × 4.6 mm, 5 μm) and a UV spectrophotometer detector operated at 225 nm. Sodium [^99m^Tc] pertechnetate Na[⁹⁹ᵐTcO₄]^⁻^ was eluted from a [⁹⁹Mo]Mo/[⁹⁹ᵐTc]Tc generator (activity: 1 Ci) supplied by the Radioisotope Production Factory (RPF), Egyptian Atomic Energy Authority. Radioactivity measurements were performed using a well-type Na-I (Tl) crystal gamma counter (USA).

### Animal Model

In this study, six-week-old Swiss Albino male mice, each weighing between 20 and 35 g, were utilized. The animals were housed in a controlled environment maintained at a temperature of 22 ± 2 °C, under a standardized 12-hour light/dark cycle. Throughout the experiment period, the mice had unrestricted access to standard laboratory chow and filtered water to ensure consistent nutritional and hydration status.

### Induction of the EAC Model

Two distinct groups of mice were used: Group I (Control): Healthy mice without tumor induction. Group II (Tumor-bearing mice. Mice were injected intramuscularly with Ehrlich ascites carcinoma (EAC) cells. Each group was subdivided into five timepoints (*n* = 7 per timepoint) for biodistribution analysis. The EAC cell line, derived from a murine mammary carcinoma, was obtained from the National Cancer Institute in Cairo, Egypt [27]. Cells were suspended in Dulbecco’s PBS (36 µg/mL C₃H₃NaO₃ and 1 mg/mL C₆H₁₂O₆) mixed with sterile Matrigel. A volume of 200 µL containing 2 × 10⁶ cells was subcutaneously injected into the right hind flank of the tumor-bearing mice. Tumor development was monitored, and uptake was confirmed when tumors reached 500–800 mm³ within one week [28].

### Radiolabeling of Tamsulosin

In evacuated vials, various amounts of tamsulosin ligand (0.2 to 1 mg) were dissolved in 0.5 mL of double-distilled water purged with nitrogen. Then, freshly prepared stannous chloride solution (25 to 150 µg) was added [29]. The pH of the solution was adjusted to different values (2–10) by adding 0.2 mL of the appropriate buffer. Finally, about 0.1 mL (370 MBq) of freshly eluted sodium [^99m^Tc] pertechnetate Na[⁹⁹ᵐTcO₄]^⁻^ was added to each vial [30]. The mixture was thoroughly mixed and incubated for 5 to 60 min at 25 °C. Radiochemical yield was assessed through thin-layer chromatography (TLC) on aluminum-silica gel plates, with quantification of radioactivity performed using a γ-scintillation counter [31].

### Factors Affecting the Radiolabeling Yield

The radiolabeling of Tamsulosin with technetium-99m was systematically optimized by assessing the effects of various parameters, such as ligand amount, reducing agent amount, pH of the reaction medium, and reaction time, to achieve a high radiochemical yield of the [^99m^Tc]Tc-Tamsulosin complex [32]. Each parameter in the experimental setup was tested in triplicate to enhance reproducibility and ensure statistical reliability. The radiochemical purity was measured using thin-layer chromatography (TLC) with aluminum-silica gel plates, and radioactivity was detected by a gamma scintillation counter [33]. To further confirm the yield and evaluate radiochemical purity, electrophoresis and high-performance liquid chromatography (HPLC) analyses were also conducted [34, 35].

### Quality Control of the [^99m^Tc]Tc-Tamsulosin Complex

#### Paper Chromatography

The TLC technique was used to determine the radiochemical yield percentage of the [^99m^Tc]Tc-Tamsulosin complex. A portion of the tracer was applied to a TLC strip, with acetone serving as the mobile phase to quantify free pertechnetate [^99m^TcO_4_]^−^. In this system, free pertechnetate [^99m^TcO_4_]^−^ moved to the solvent front (Rf = 1), while the [^99m^Tc]Tc-Tamsulosin complex and the reduced hydrolyzed technetium [^99m^Tc] (RH-^99m^Tc, colloids) remained at the application point. To assess the quantity of the reduced hydrolyzed technetium [^99m^Tc] (RH-^99m^Tc, colloids), a mobile phase composed of NH_4_OH, ethanol (EtOH), and water (H_2_O) as a mixture in a 1:2:5 (v/v/v) ratio was employed [36]. In this setup, the colloid stayed at the origin (Rf = 0), while other species moved toward the solvent front (Rf = 1) [37]. Finally, the strips were allowed to dry in the air for a while and were divided into 1 cm segments to measure their radioactivity. The radiochemical yield percentage of the complex was calculated by subtracting the amounts of colloid and free pertechnetate from 100%.

#### Electrophoresis Purification

Cellulose acetate strips were used to determine the radiochemical yield percentage of the [^99m^Tc]Tc-Tamsulosin complex, confirming the TLC results. The strips were moistened with a 0.9 N NaCl solution before being placed in the chamber. The [^99m^Tc]Tc-Tamsulosin complex was filtered through a 0.22 μm Millipore filter [38]. A 10 µl aliquot of the tracer was applied 6 cm from the cathode. Electrophoresis was performed for 1.5 h at 300 volts [39]. Afterward, the strips were removed, cut into 1 cm pieces, and their radioactivity was measured using a gamma counter. The radiochemical yield percentage was then calculated.

#### HPLC Purification

To evaluate the radiochemical purity of the [^99m^Tc]Tc-Tamsulosin complex, high-performance liquid chromatography (HPLC) was employed. A sample volume of 20 µL of the [^99m^Tc]Tc-Tamsulosin complex was injected into the system. Chromatographic separation was achieved using a reverse-phase C18 column (dimensions: 25 cm × 4.6 mm, particle size: 5 μm. The mobile phase consisted of a binary gradient system: solution A was an aqueous buffer of potassium dihydrogen phosphate (KH₂PO₄) at a concentration of 0.05 mM, adjusted to pH 3.5 to optimize retention and peak symmetry; solution B was acetonitrile, an organic solvent chosen for its compatibility with UV detection [40]. The ratio of solution A to B was maintained at 55:45 (v/v), ensuring adequate separation of the [^99m^Tc]Tc-Tamsulosin complex from potential impurities or degradation products. The flow rate was set at 1.0 mL/min, and all analyses were conducted at ambient room temperature to maintain consistent chromatographic conditions and preserve compound stability. Eluted fractions were collected in 1.0 mL aliquots using an automated fraction collector, where each collected fraction was subsequently subjected to gamma counting to assess radiochemical purity and confirm the integrity of the radiotracer. The same protocol was adapted for cold tamsulosin to evaluate the chemical purity and chromatographic behavior of cold (inactive) tamsulosin. High-performance liquid chromatography (HPLC) was employed with ultraviolet (UV) detection at a wavelength of 225 nm, corresponding to the absorbance maximum of the tamsulosin chromophore.

#### Lipophilicity

The lipophilicity of the [^99m^Tc]Tc-Tamsulosin complex was evaluated by its partition coefficient (P), determined through its distribution between 1-octanol and phosphate buffer. A 100 µL volume of the tracer was vortexed with 500 µL of the biphasic system, then centrifuged and measured in triplicate [41]. Radioactivity in each phase was quantified using a gamma counter, and P was expressed as the logarithm of the ratio of counts in the organic phase to the aqueous phase.

#### In Vitro Stability Study

The stability of the [^99m^Tc]Tc-Tamsulosin complex was assessed in both fresh serum and phosphate-buffered saline (PBS) at various times. A mixture of 100 µl of [^99m^Tc]Tc-Tamsulosin complex and 1 ml of either fresh serum or PBS (pH 7.4) was prepared. At each designated interval, 10 µL of this mixture was examined using a TLC technique [42].

#### In Vivo Evaluation Study

The in vivo evaluation of the [^99m^Tc]Tc-Tamsulosin complex was conducted at various time intervals (*n* = 7). Initially, the [^99m^Tc]Tc-Tamsulosin complex was filtered through a 0.22 μm Millipore membrane to remove colloidal impurities. Subsequently, both tumor-bearing and normal mice were administered 0.2 mL of the [^99m^Tc]Tc-Tamsulosin complex (50–100 MBq) intravenously via the tail vein. At predetermined time intervals, the mice were anesthetized, weighed, and sacrificed at 0.5, 1, 1.5, 2, and 3 h post-injection (p.i.). All body organs and tissues were excised, rinsed, dried, and weighed [43]. The radioactivity of all organs, as well as the background, was measured using a γ-counter. The percent injected dose per gram of tissue (%ID/g) p.i. was calculated at each time point and expressed as mean ± SD. The target (solid tumor) to non-target (normal muscle) ratio (T/NT) was determined from the percentage of injected dose per gram (% ID/g) for the solid tumor compared to normal muscle.

### Statistical Analysis

All data were analyzed using a one-way analysis of variance. P values are reported, and all data were expressed as mean ± SEM. Statistical significance was set at *p* < 0.05.

## Results

### Radiolabeling of the [^99m^Tc]Tc-Tamsulosin Complex

**Fig. 2:**
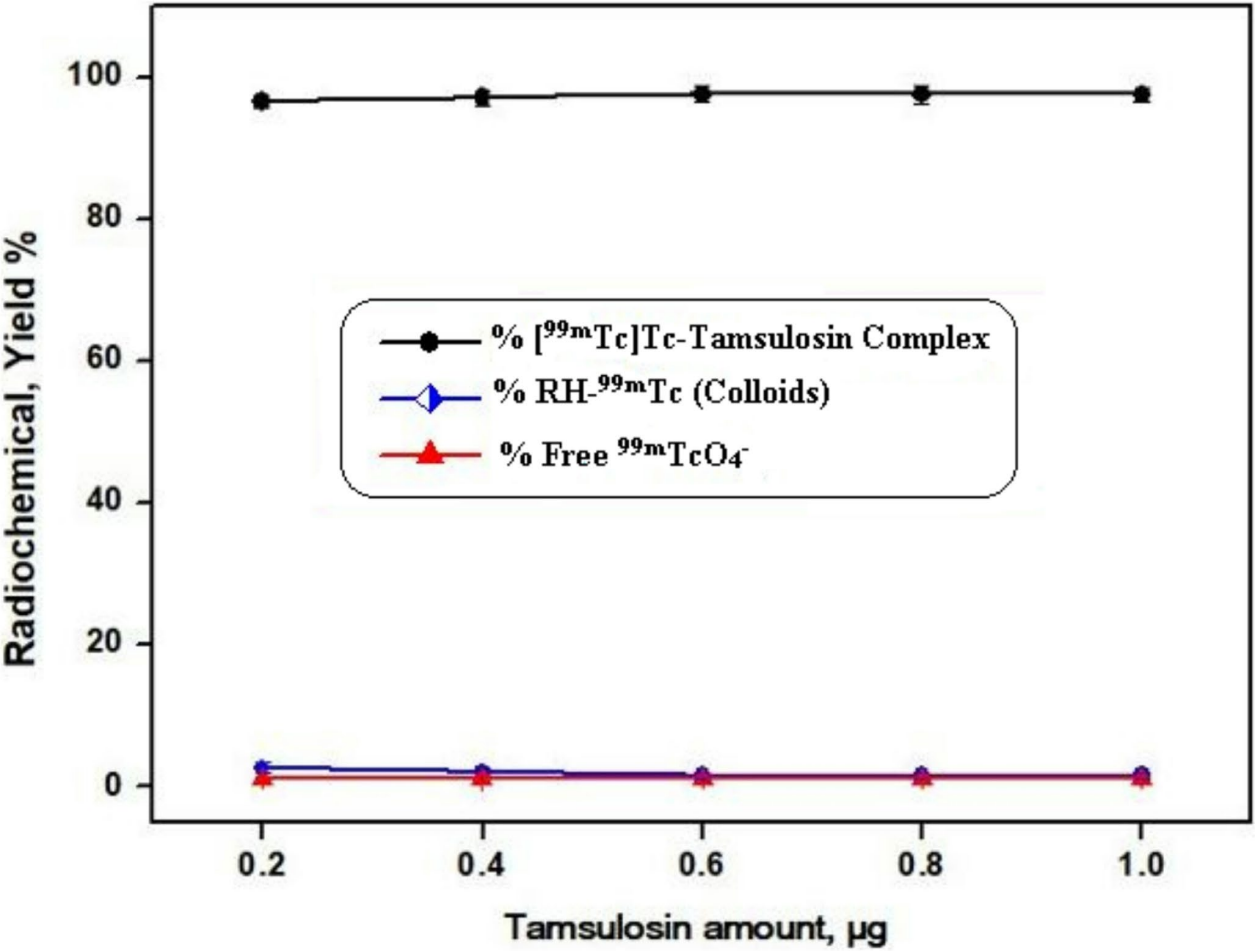
The radiochemical yield % of [^99m^Tc]Tc-Tamsulosin complex versus the Tamsulosin amount. Reaction conditions: X mg of Tamsulosin,75 µg of SnCl_2_. 2H_2_O, 100 µL of [^99m^TcO_4_]^-^ solution (7.2 MBq), pH 4, at room temperature for 30 min

**Fig. 3:**
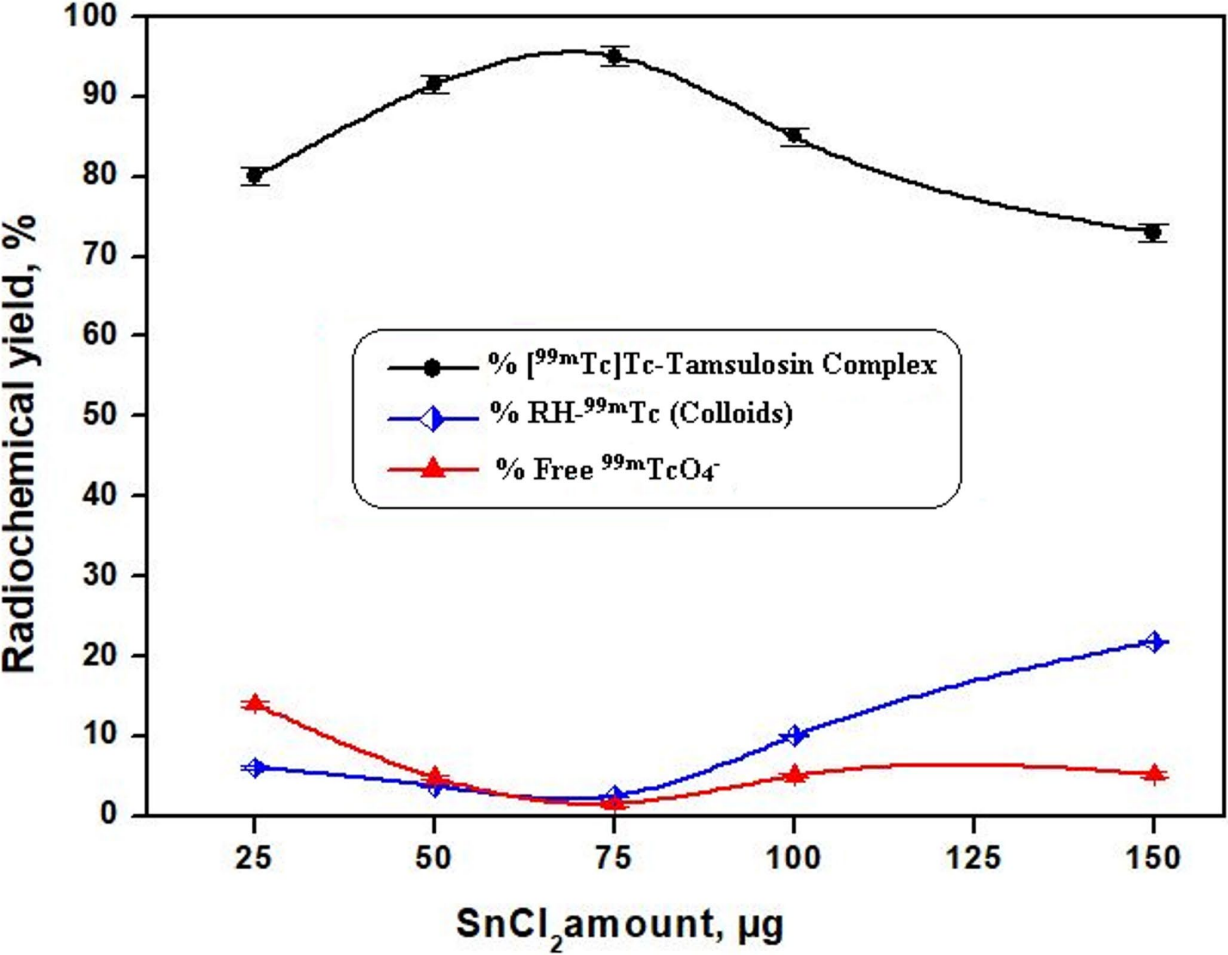
The radiochemical yield % of [^99m^Tc]Tc-Tamsulosin complex versus SnCl_2_.2H_2_O amount. Reaction conditions: 0.6 mg of Tamsulosin, X µg of SnCl_2_.2H_2_O solution, 100 µL of [^99m^TcO_4_]^-^ solution (7.2 MBq), pH 4, at room temperature for 30 min

**Fig. 4:**
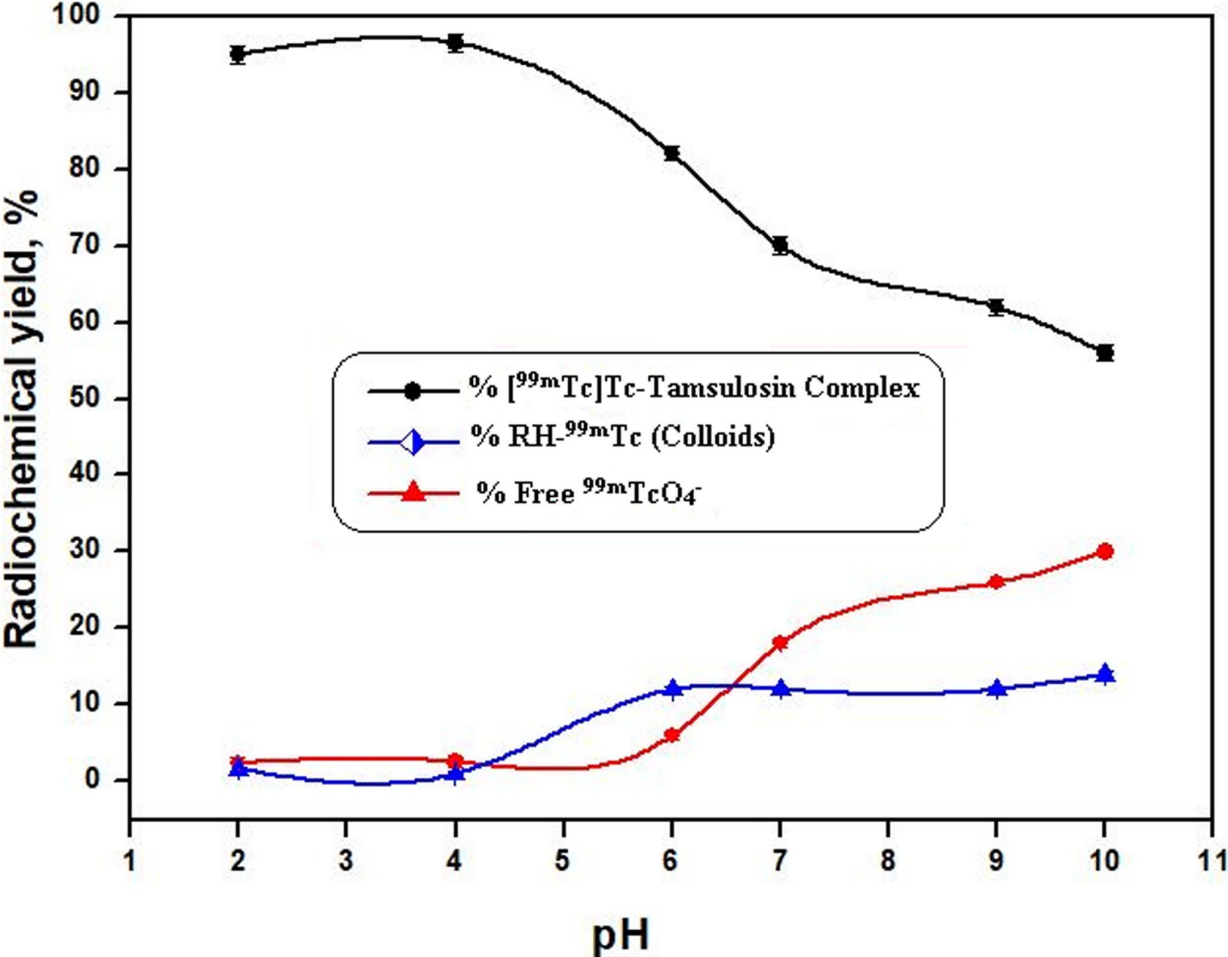
The radiochemical yield % of [^99m^Tc]Tc-Tamsulosin complex versus reaction pH. Reaction conditions: 0.6 mg of Tamsulosin, 75 µg of SnCl_2_. 2H_2_O solution, 100 µL of [^99m^TcO_4_]^-^ solution (7.2 MBq), 200 µL buffer of different pH (2–10), at room temperature for 30 min

**Fig. 5:**
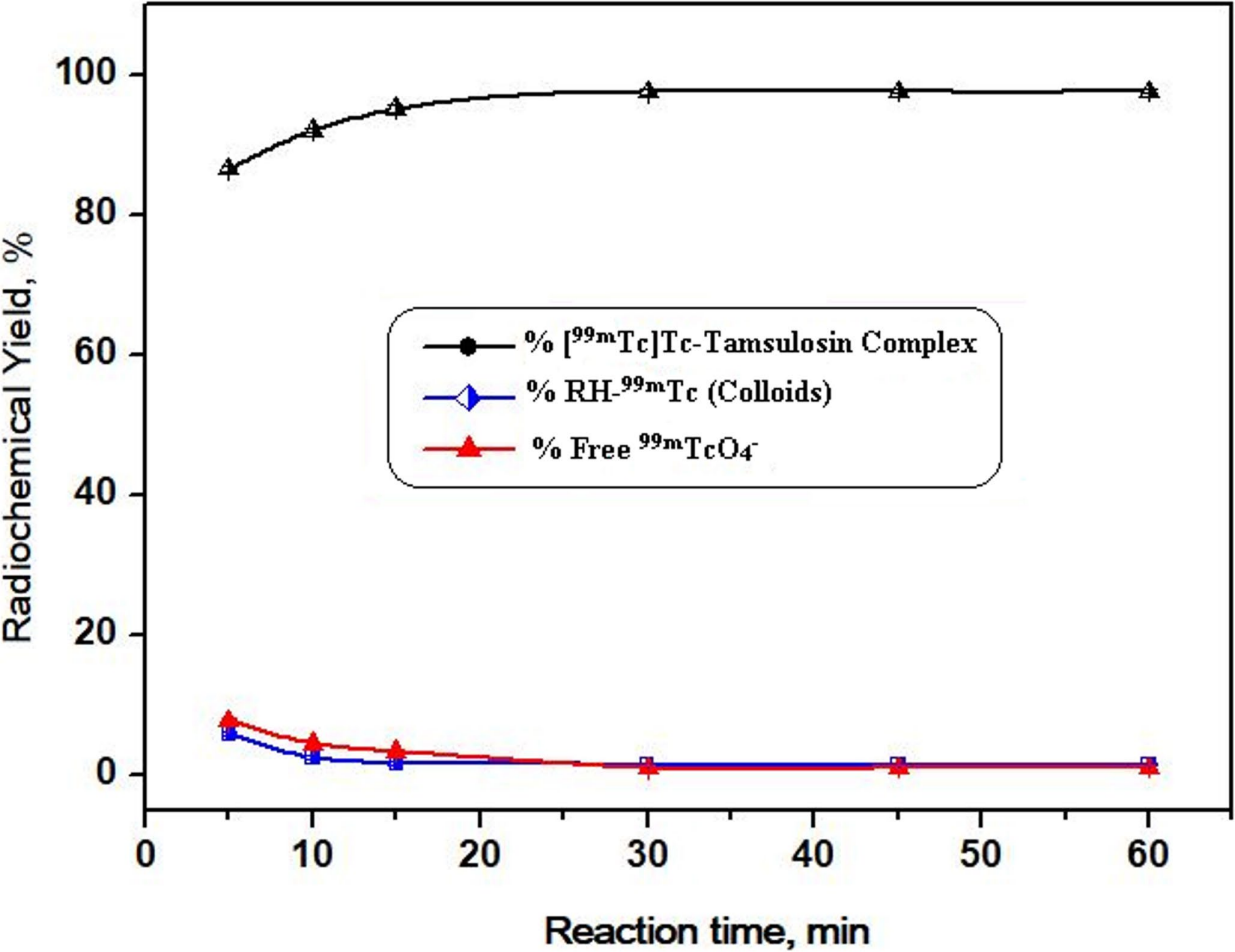
The radiochemical yield % of [^99m^Tc]Tc-Tamsulosin complex versus reaction time. Reaction conditions: 0.6 mg of Tamsulosin, 75 µg of SnCl_2_. 2H_2_O solution, 100 µL of [^99m^TcO_4_]^-^ solution (7.2 MBq), 200 µL buffer of pH 4, at room temperature for various times

The proposed coordination structure of tamsulosin with technetium-99m is depicted in Fig. 1b. The optimal radiochemical yield of the [^99m^Tc]Tc-Tamsulosin complex was determined to be 97.5 ± 0.12% under conditions involving 0.6 mg of Tamsulosin and 75 µg of stannous chloride. The synthesis was performed at 25 °C for 30 min, with a pH of 4. Figures 2, 3, 4 and 5 illustrate all factors influencing the radiochemical yield of the [^99m^Tc]Tc-Tamsulosin complex. The data reveal that an optimal radiochemical yield of 94.5 ± 1.1% was achieved with a tamsulosin amount of 0.6 mg, as shown in Fig. 2. Beyond this quantity, no further enhancement in radiochemical yield was observed. The study examined the role of stannous chloride dihydrate as a reductant in the chelation of technetium-99m with tamsulosin, as depicted in Fig. 3. At a low concentration of 25 µg, SnCl_2_·2H_2_O facilitated a radiochemical yield of 80 ± 1.3%. Increasing the amount to 75 µg resulted in a peak yield of 96 ± 1.1%. Conversely, an excess of SnCl_2_·2H_2_O at 150 µg led to a decrease in radiochemical yield of the [^99m^Tc]Tc-Tamsulosin complex to 73 ± 1.3% and an increase in colloid formation (reduced hydrolyzed technetium, RH-^99m^Tc) to 21.8 ± 0.7%.

The investigation of the radiochemical yield of the [^99m^Tc]Tc-Tamsulosin complex across a pH range of 2 to 10 is shown in Fig. 4. At pH 2, the radiochemical yield was relatively high, measuring 95 ± 0.8%. However, the highest radioactivity was observed at pH 4, with a yield of 96.5 ± 0.7%. As the pH increased further, a noticeable change in the yield pattern occurred. The radiochemical yield dropped significantly to 56 ± 0.8% at pH 10, while the colloidal technetium yield increased to 30 ± 0.5%.

The effect of reaction time on the radiochemical yield of the [^99m^Tc]Tc-Tamsulosin complex is shown in Fig. 5. It is observed that as reaction time increases, the radiochemical yield also increases, reaching a maximum of 97.5 ± 1.0% at 30 min. Extending the reaction time beyond 30 min does not lead to a further increase in yield.

### Quality Control of the [^99m^Tc]Tc-Tamsulosin Complex

**Fig. 6:**
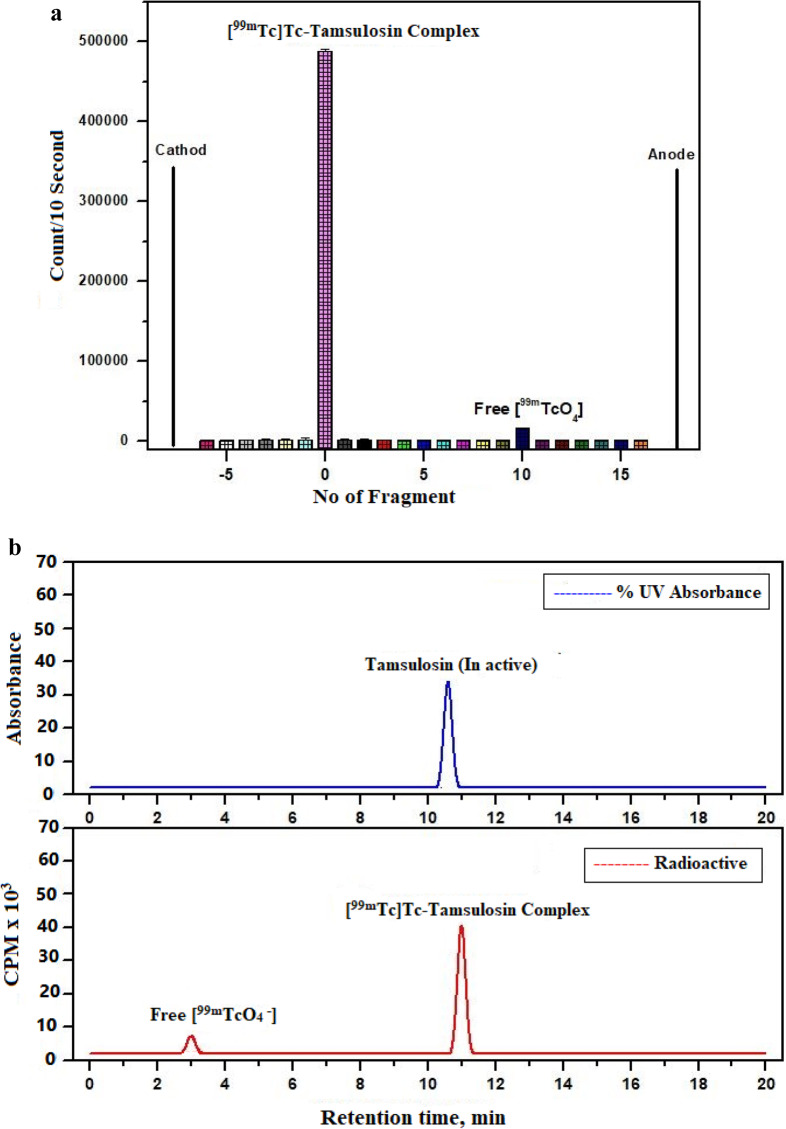
**a, b.** Quality control of the [^99m^Tc]Tc-Tamsulosin complex by electrophoresis and HPLC. (**a**) Electrophoresis radiochromatogram of [^99m^Tc]Tc-Tamsulosin complex. (**b**) HPLC radiochromatogram of the [^99m^Tc]Tc-Tamsulosin complex: HPLC showed separation of free technetium-99m [^99m^TcO_4_]^-^ and [^99m^Tc]Tc-Tamsulosin complex, which were given at Rt of 3 and 11 min, respectively; HPLC-UV of Tamsulosin was given at Rt = 10.5 min

The radiochemical purity of the synthesized [⁹⁹ᵐTc]Tc-Tamsulosin complex was assessed using various analytical techniques. Thin-layer chromatography (TLC) demonstrated a radiochemical yield of 97.5 ± 0.12%. Subsequent purification through electrophoresis confirmed a similar purity level of 97.5 ± 1.1%, as depicted in Fig. 6a. High-performance liquid chromatography (HPLC) analysis supported these results, indicating a radiochemical purity of 97.5 ± 1.0%. The HPLC chromatogram displayed distinct retention times of 3 min for free pertechnetate [TcO₄]⁻ and 11 min for the [⁹⁹ᵐTc]Tc-Tamsulosin complex. Furthermore, UV spectrophotometric analysis of the non-radioactive tamsulosin compound showed a maximum absorbance at 10.5 min with a wavelength of 225 nm, as illustrated in Fig. 6b.

### In Vitro Stability and Lipophilicity

**Fig. 7:**
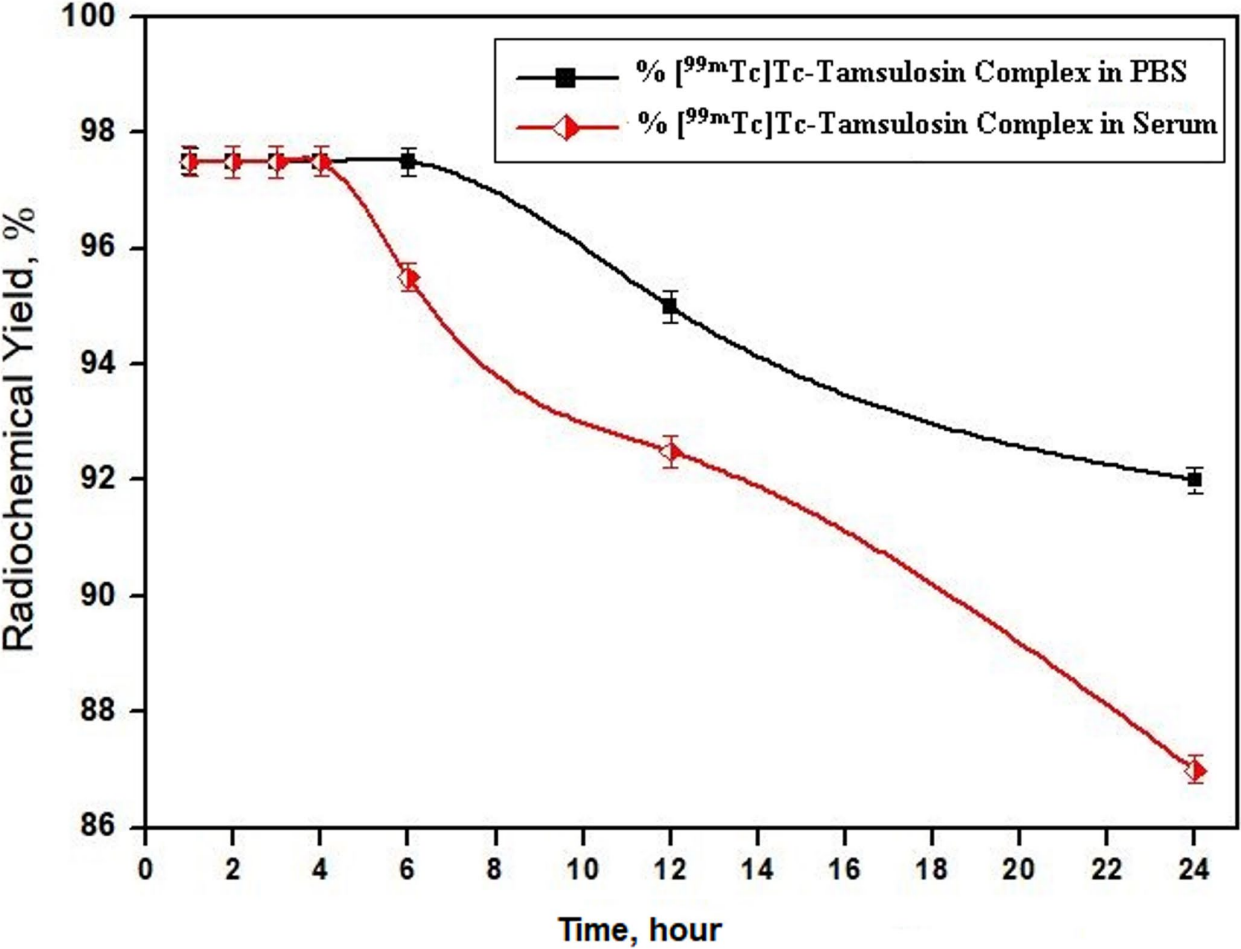
In vitro Stability of [^99m^Tc]Tc-Tamsulosin complex in fresh serum and PBS over various times

The stability of the [^99m^Tc]Tc-Tamsulosin complex in vitro in serum compared to PBS is shown in Fig. 7. In serum, the stability decreased from 97.5 ± 0.5% to 87 ± 0.6% (approximately 9.5%) after 24 h, whereas in PBS, it declined from 97.5 ± 0.4% to 92 ± 0.3% (approximately 4.5%) over the same period. Analysis of the lipophilicity of the [^99m^Tc]Tc-Tamsulosin complex revealed that the observed partition coefficient (P) was 1.85 ± 0.03%, categorizing it as a moderately lipophilic compound.

### In Vivo Evaluation Results

In vivo evaluation studies of the [⁹⁹ᵐTc]Tc-Tamsulosin complex were conducted using a normal mouse model, as detailed in Table 1. The data indicated rapid systemic circulation, with peak radioactivity observed at 0.5 h post-injection at 6.9 ± 0.18% ID/g. Subsequently, a decline in blood radioactivity was noted, reaching 1.8 ± 0.11% ID/g at 3 h post-injection. Notably, the tracer demonstrated significant uptake in the kidneys, with values of 14 ± 0.35% ID/g at 2 h, corroborated by high radioactivity levels in urine, measured at 22 ± 1.4% ID/g at 3 h. Additionally, the uptake of the [⁹⁹ᵐTc]Tc-Tamsulosin complex in the liver was recorded at 4.8 ± 0.11%ID/g at 2 h, while its presence in the intestine was 5.3 ± 0.14% ID/g at the same time point. Furthermore, normal uptake of the [⁹⁹ᵐTc]Tc-Tamsulosin complex was observed in muscle tissue, with values of 0.98 ± 0.02% ID/g, 1.1 ± 0.02% ID/g, 0.95 ± 0.01% ID/g, 0.8 ± 0.01% ID/g, and 0.6 ± 0.01% ID/g at 0.5, 1, 1.5, 2, and 3 h post-injection, respectively. Conversely, minimal accumulation was observed in the brain, with levels reaching 0.46 ± 0.05% ID/g at 0.5 h, decreasing to 0.09 ± 0.01% ID/g at 3 h post-injection. A normal distribution was also observed in the bone, lung, and heart.

**Table 1:** Biodistribution of the [⁹⁹ᵐTc]Tc-Tamsulosin complex in a normal mouse model. Values are represented as %ID/g tissue, and expressed as mean ± SD, *n* = 7

Organs/Tissues	[^99m^Tc]Tc-Tamsulosin complex in a normal mouse model, % ID/g tissue				
	Time post injection (h.)				
	0.5 h.	1 h.	1.5 h.	2 h.	3 h.
Blood	6.9 ± 0.18	4.5 ± 0.16	3.2 ± 0.15	2.4 ± 0.14	1.8 ± 0.11
Bone	0.22 ± 0.01	0.20 ± 0.01	0.11 ± 0.01	0.08 ± 0.01	0.06 ± 0.01
Muscles	0.98 ± 0.02	1.1 ± 0.02	0.95 ± 0.01	0.8 ± 0.01	0.6 ± 0.01
Brain	0.46 ± 0.05	0.33 ± 0.04	0.28 ± 0.02	0.16 ± 0.02	0.12 ± 0.01
Stomach	3.3 ± 0.12	4.8 ± 0.11	3.2 ± 0.14	2.8 ± 0.16	1.5 ± 0.11
Intestine	1.6 ± 0.11	3.9 ± 0.12	4.2 ± 0.11	5.3 ± 0.14	2.2 ± 0.11
Kidney	3.8 ± 0.30	6.5 ± 0.32	11.5 ± 0.22	14 ± 0.35	9.5 ± 0.26
Liver	1.8 ± 0.22	2.5 ± 0.12	4.2 ± 0.12	4.8 ± 0.11	2.8 ± 0.10
Spleen	1.2 ± 0.03	1.4 ± 0.03	1.8 ± 0.04	1.87 ± 0.03	0.72 ± 0.02
Lung	1.9 ± 0.08	1.7 ± 0.04	1.5 ± 0.03	1.1 ± 0.02	0.85 ± 0.03
Heart	3.4 ± 0.14	1.8 ± 0.11	1.5 ± 0.06	1.2 ± 0.04	0.92 ± 0.02
Urine	2.7 ± 0.11	7.2 ± 1.20	10 ± 1.22	16 ± 1.35	22 ± 1.4
Stool	-	-	1.6 ± 0.08	-	2.4 ± 1.12

**Fig. 8:**
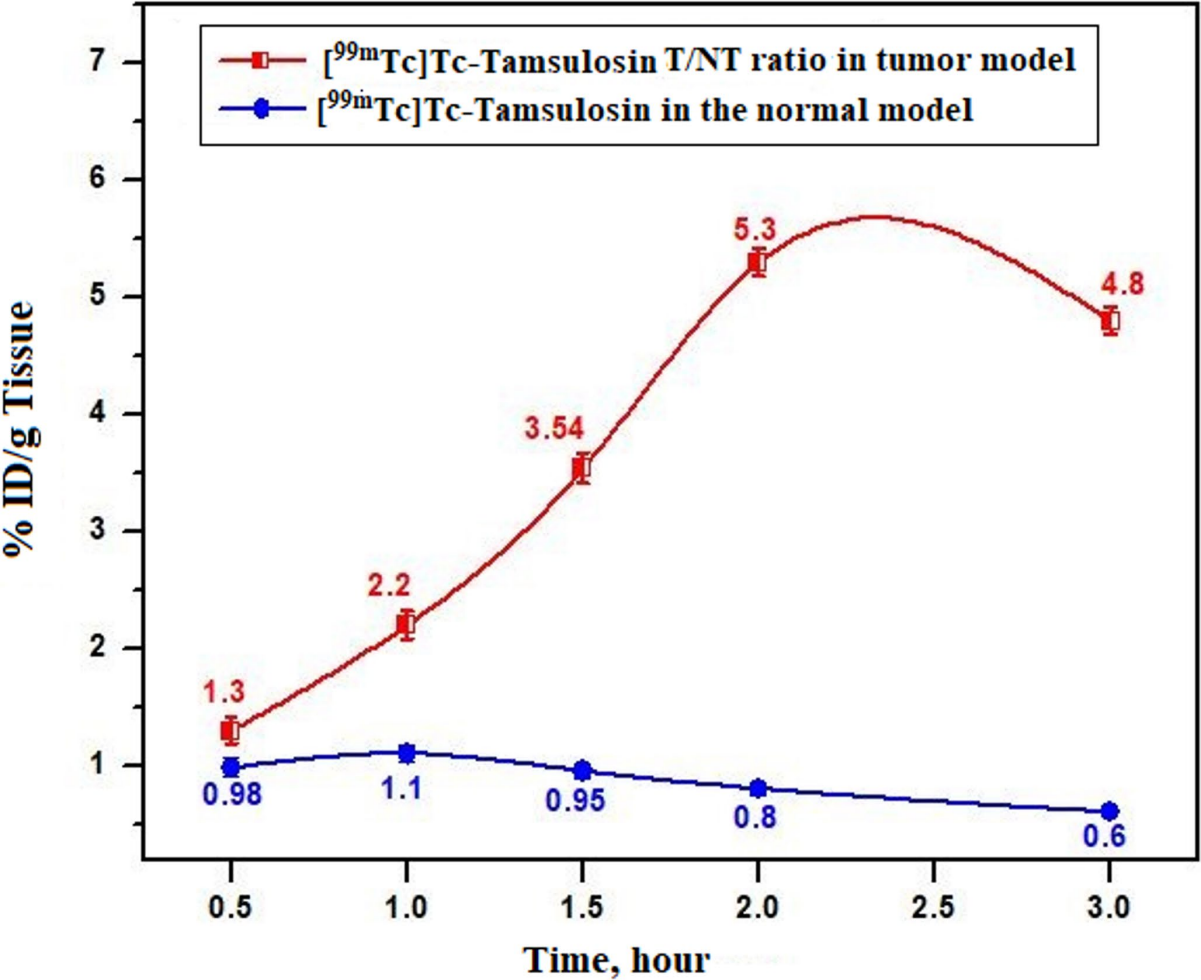
Ratio uptake (T/NT) of [^99m^Tc]Tc-Tamsulosin complex in tumor-bearing compared to normal mice versus time

In vivo evaluation studies of the [⁹⁹ᵐTc]Tc-Tamsulosin complex in mice bearing a solid tumor model are presented in Table 2. The initial rapid distribution of the [⁹⁹ᵐTc]Tc-Tamsulosin complex in the bloodstream was observed, reaching 7.5 ± 0.19% ID/g at 0.5 h post-injection, followed by a decline to 2.5 ± 0.16% ID/g at 3 h post-injection. The data indicated significant uptake in the liver and kidneys, with values of 5.5 ± 0.22% ID/g and 16 ± 0.24% ID/g at 2 h post-injection, respectively. Conversely, minimal accumulation was noted in the brain, with levels reaching 0.53 ± 0.06% ID/g at 0.5 h and decreasing to 0.25 ± 0.04% ID/g at 3 h post-injection. Normal distribution was observed in the bone, lung, and heart. Notably, the [⁹⁹ᵐTc]Tc-Tamsulosin complex demonstrated a pronounced and selective accumulation in tumor tissue, achieving peak uptake of 5.2 ± 0.02% ID/g at 2 h post-injection, significantly exceeding non-target tissue uptake (0.98 ± 0.01% ID/g), with a target-to-non-target (T/NT) ratio of 5.3 at 2 h post-injection, as illustrated in Fig. 8.

**Table 2:** Biodistribution of the [⁹⁹ᵐTc]Tc-Tamsulosin complex in mice bearing a solid tumor model values are represented as %ID/g tissue, and expressed as mean ± SD, *n* = 7

Organs/Tissues	[^99m^Tc]Tc-Tamsulosin complex in mice bearing a solid tumor model, ID/g tissue				
	Time post injection (h.)				
	0.5 h.	1 h.	1.5 h.	2 h.	3 h.
Blood	7.5 ± 0.19	5.7 ± 0.18	4.4 ± 0.17	3.8 ± 0.15	2.5 ± 0.16
Bone	0.21 ± 0.02	0.18 ± 0.02	0.12 ± 0.02	0.09 ± 0.01	0.07 ± 0.01
Tumor (T)	1.3 ± 0.03	2.6 ± 0.02	3.9 ± 0.03	5.2 ± 0.02	4.6 ± 0.02
Muscles (NT)	1.0 ± 0.01	1.2 ± 0.01	1.1 ± 0.02	0.98 ± 0.01	0.95 ± 0.01
Brain	0.53 ± 0.06	0.42 ± 0.06	0.37 ± 0.07	0.31 ± 0.05	0.25 ± 0.04
Stomach	3.1 ± 0.11	4.8 ± 0.12	3.3 ± 0.17	3.1 ± 0.18	1.9 ± 0.11
Intestine	1.8 ± 0.10	4.6 ± 0.12	4.8 ± 0.12	5.5 ± 0.15	2.2 ± 0.11
Kidney	3.6 ± 0.20	5.2 ± 0.22	10.8 ± 0.25	16 ± 0.24	10 ± 0.25
Liver	2.2 ± 0.11	3.7 ± 0.14	4.7 ± 0.20	5.5 ± 0.22	3.1 ± 0.11
Spleen	1.1 ± 0.04	1.3 ± 0.04	1.7 ± 0.03	1.9 ± 0.03	0.85 ± 0.02
Lung	2.1 ± 0.02	1.8 ± 0.03	1.7 ± 0.01	1.2 ± 0.01	0.78 ± 0.01
Heart	3.2 ± 0.15	2.2 ± 0.14	1.4 ± 0.12	1.3 ± 0.11	0.98 ± 0.07
Urine	3.5 ± 0.12	6.5 ± 1.11	8.5 ± 1.15	12 ± 1.26	25 ± 1.37
Stool	-	-	-	2.5 ± 0.12	3.8 ± 0.15
T/NT	**1.3**	**2.2**	**3.54**	**5.3**	**4.8**

## Discussion

Tamsulosin hydrochloride, formally designated as (R)−5-[2-[[2-(o-Ethoxyphenoxy) ethyl] amino] propyl]−2-methoxybenzenesulfonamide hydrochloride, exhibits promising characteristics as a ligand for technetium-99 m [⁹⁹ᵐTc] complexation [44]. Successful radiolabeling with technetium-99 m typically requires the presence of functional groups capable of chelating the metal ion, particularly when technetium-99 m is reduced from its native [+ VII] oxidation state as pertechnetate, [⁹⁹ᵐTcO₄⁻], to lower oxidation states (+ V,+IV, or + III) using reducing agents such as stannous chloride. These reduced forms exhibit enhanced coordination chemistry, rendering them more suitable for incorporation into radiopharmaceutical compounds. The molecular architecture of tamsulosin contains several functional moieties conducive to metal coordination. Notably, the sulfonamide group (–SO₂NH–) offers both nitrogen and oxygen donor atoms, while the secondary amine (–NH–) serves as a Lewis base through its lone electron pair. Additional oxygen-donating groups, including ether linkages (–O–), methoxy (–OCH₃), and phenoxy functionalities, further augment the ligand’s chelation potential [45].

Collectively, these features suggest that tamsulosin possesses the requisite structural attributes for stable and efficient complexation with technetium-99 m, thereby supporting its utility in radiopharmaceutical development. Structural analysis suggests that the most likely binding sites for [⁹⁹ᵐTc] are the deprotonated sulfonamide nitrogen, which functions as an anionic donor, and the secondary amine nitrogen within the ethylamino chain, which acts as a neutral donor [46]. These groups may collectively form stable metal–nitrogen bonds, resulting in a chelated structure that integrates the benzene ring, the propyl chain, and the ethylamino linker into a flexible coordination framework [47]. The coordination sphere of technetium may be further completed by additional ligands, such as water molecules or chloride ions from the hydrochloride salt, potentially forming a stable tetrahedral complex suitable for imaging applications, as illustrated in Fig. 1b.

Following the radiolabeling process, the mobile phase comprised acetone and a mixture of NH_4_OH, EtOH, and H_2_O in a 1:2:5 (v/v/v) ratio, facilitating the assessment of the radiochemical yield percentage of the [^99m^Tc]Tc-Tamsulosin complex. The resulting species can be categorized into three primary fractions: (i) the desired [^99m^Tc]Tc-Tamsulosin complex, which is the radiolabeled compound; (ii) colloidal technetium, also known as reduced hydrolyzed technetium (RH-^99m^Tc) or “colloids,” which are unwanted by-products formed during hydrolysis; and (iii) free pertechnetate [^99m^TcO_4_]^−^, representing unbound technetium [48–50]. The relative proportions of these fractions are routinely determined to assess the radiochemical purity and yield of the [^99m^Tc]Tc-Tamsulosin complex using ascending paper chromatography (TLC) [51].

A systematic analysis was conducted on various parameters affecting the radiochemical yield, including the quantity of tamsulosin (ligand), the amount of reducing agent, pH, and incubation time [52]. Each parameter within the experimental setup was tested in triplicate to enhance reproducibility and ensure statistical reliability. The radiochemical yield of the [⁹⁹ᵐTc]Tc-Tamsulosin complex is significantly affected by the concentration of tamsulosin hydrochloride. A maximum yield of 94.5 ± 1.1% was achieved at a ligand concentration of 0.6 mg, indicating this concentration as the optimal quantity for efficient complexation. Concentrations below this threshold resulted in suboptimal labeling, likely due to insufficient ligand availability for effective chelation. Conversely, increasing the ligand concentration beyond 0.6 mg did not further enhance the yield, suggesting a saturation point in the coordination process [53].

Similarly, the concentration of the reducing agent, stannous chloride dihydrate (SnCl₂·2 H₂O), was critical in determining the radiochemical yield. An optimal yield of 96 ± 1.1% was observed at 75 µg, reflecting efficient reduction of pertechnetate and subsequent chelation with the ligand. However, both lower (25 µg) and higher (150 µg) concentrations resulted in diminished yields of 80 ± 1.3% and 73 ± 1.3%, respectively, accompanied by a notable increase in colloid formation (21.8 ± 0.7%). This inverse relationship suggests that excessive SnCl₂·2 H₂O may promote side reactions, including the complete reduction of pertechnetate to insoluble technetium dioxide colloid species [TcO₂·xH₂O], thereby impeding effective complexation with Tamsulosin [54]. Moreover, the pH of the reaction medium was observed to have a substantial effect on the radiolabeling efficiency. The optimal radiochemical yield (96.5 ± 0.7%) was achieved at pH 4, with similarly high efficiency noted at pH 2 (95 ± 0.8%). Beyond this acidic range, particularly under alkaline conditions, the yield decreased significantly. This decline is attributed to increased colloid formation, likely due to stannous chloride precipitation (formation of reduced Tin particles), and insoluble reduced hydrolyzed technetium (RH-^99m^Tc), colloid species may be formed as undesired by-products [55]. The radiochemical yield of the [⁹⁹ᵐTc]Tc-Tamsulosin complex was determined to be time-dependent, with a progressive increase observed up to 30 min, at which point a maximum yield of 97.5 ± 1.0% was achieved. No significant enhancement was noted beyond this duration, indicating that 30 min represents the optimal reaction time for complete complexation of Tamsulosin with technetium-99m under the studied conditions. Optimal radiolabeling conditions were established using 0.6 mg of tamsulosin hydrochloride and 75 µg of stannous chloride at pH 4 and a temperature of 25 °C, with a reaction time of 30 min. These parameters yielded a maximum radiochemical yield of 97.5 ± 0.12%, as depicted in Figs. 2, 3, 4, and 5.

Quality control of the [⁹⁹ᵐTc]Tc-Tamsulosin complex was meticulously evaluated using electrophoresis and high-performance liquid chromatography (HPLC), both of which confirmed substantial radiochemical integrity. Electrophoretic analysis indicated a radiochemical yield of approximately 97.5 ± 1.1%, with the [⁹⁹ᵐTc]Tc-Tamsulosin complex remaining at the origin (fragment number zero). At the same time, free pertechnetate [⁹⁹ᵐTcO₄]⁻ migrated toward the anode, appearing at fragment number ten (Fig. 6a). This distinct separation underscores the high purity of the radiolabeled complex and the minimal presence of unbound pertechnetate, indicating robust tracer stability. HPLC analysis further confirmed the radiochemical purity, yielding a value of 97.5 ± 1.0%. The chromatographic profile demonstrated clear resolution between free pertechnetate [⁹⁹ᵐTcO₄]⁻ and the labeled complex [⁹⁹ᵐTc]Tc-Tamsulosin complex, with retention times of 3 min and 11 min, respectively. These findings confirm the successful formation and isolation of the [⁹⁹ᵐTc]Tc-Tamsulosin complex. Additionally, UV spectrophotometric analysis of the non-radioactive Tamsulosin compound revealed a maximum absorbance at 10.5 min at a wavelength of 225 nm (Fig. 6b), consistent with the expected chromatographic behavior of the parent molecule. Collectively, these results affirm the chemical and radiochemical stability of the complex and support its suitability for further biological evaluation.

Stability assessments of the [⁹⁹ᵐTc]Tc-Tamsulosin complex in phosphate-buffered saline (PBS) and serum media revealed an initial radiochemical integrity of approximately 97.5 ± 0.5%. However, a gradual decline in stability was observed over 24 h, with the complex exhibiting greater degradation in serum (87 ± 0.4%) compared to PBS (92 ± 0.6%), as shown in Fig. 7. Despite this reduction, the complex maintained acceptable stability in both media, indicating its suitability for in vivo applications [56]. Furthermore, the [⁹⁹ᵐTc]Tc-Tamsulosin complex demonstrated a partition coefficient (P) of 1.85 ± 0.03, indicative of moderate lipophilicity. This physicochemical property is advantageous for membrane permeability and may facilitate enhanced cellular uptake, thereby supporting the complex’s potential utility in targeted molecular imaging applications [57].

### In Vivo Evaluation Studies

In vivo evaluation study of the [⁹⁹ᵐTc]Tc-Tamsulosin complex was assessed in a normal mouse model, as detailed in Table 1, revealing a peak blood activity of 6.9 ± 0.18% ID/g at 0.5 h post-injection. This early peak is indicative of the rapid systemic distribution. The subsequent decrease in blood activity over time suggests efficient clearance, primarily through renal excretion and tissue uptake mechanisms. Renal accumulation of the complex reached 14 ± 0.35% ID/g at 2 h post-injection, accompanied by significant urinary excretion (22 ± 1.4% ID/g at 3 h), indicating that glomerular filtration is the principal route of elimination [58–60]. Additional uptake in the liver (4.8 ± 0.11% ID/g), spleen (1.87 ± 0.03% ID/g), and intestines (5.3 ± 0.14% ID/g) at 2 h post-injection suggests a secondary hepatobiliary clearance pathway, potentially mediated by cytochrome P450 enzymatic activity and biliary excretion [61]. Muscle tissue exhibited relatively stable uptake across all time points, implying a lack of specific binding and passive distribution through systemic circulation. Notably, brain uptake remained minimal, consistent with the moderate lipophilicity of the [⁹⁹ᵐTc]Tc-Tamsulosin complex, which allows limited penetration of the blood-brain barrier. Furthermore, distribution in bone, lung, and heart tissues was within normal physiological ranges, indicating minimal nonspecific binding and favorable pharmacokinetics.

The in vivo biodistribution study of the [⁹⁹ᵐTc]Tc-Tamsulosin complex was assessed in mice bearing a solid tumor model, as detailed in Table 2. The radiotracer demonstrated a peak blood activity of 7.5 ± 0.19% ID/g at 0.5 h post-injection, indicating rapid systemic circulation typical of low-molecular-weight radiotracers, which are designed for swift access to target tissues. This initial peak was followed by a significant decline in blood activity to 2.5 ± 0.16% ID/g at 3 h post-injection, reflecting effective systemic clearance and contributing to enhanced imaging contrast. At 2 h post-injection, notable uptake was observed in the kidneys (16 ± 0.24% ID/g), liver (5.5 ± 0.22% ID/g), spleen (1.9 ± 0.03% ID/g), and intestines (5.5 ± 0.15% ID/g), suggesting predominant renal excretion and hepatic metabolism, potentially mediated by cytochrome P450 enzymatic pathways [62]. The compound’s moderate lipophilicity, as evidenced by a log P value of 1.85 ± 0.03, appears to play a critical role in its pharmacokinetic and biodistribution characteristics. Although lipophilic agents are typically subject to uptake by the reticuloendothelial system (RES), particularly within hepatic and splenic compartments, the [⁹⁹ᵐTc]Tc-Tamsulosin complex demonstrated only modest hepatic accumulation and minimal splenic retention [63]. These findings imply that RES-mediated clearance is not the predominant elimination mechanism for this tracer. Instead, the compound exhibits favorable renal clearance kinetics, with substantial urinary excretion observed at 3 h post-injection. This balance between membrane permeability and hydrophilic elimination supports effective tumor targeting while minimizing non-specific RES sequestration.

The tracer is predominantly excreted through the renal pathway, with its accumulation in the bladder indicative of ongoing filtration and urinary elimination rather than retention. The bladder functions as a reservoir for excreted urine, and as renal clearance progresses, the concentration of radioactivity in the bladder increases cumulatively over time. This phenomenon is particularly observable in both normal and tumor-bearing mice, where bladder activity (% ID/g) escalates from 2.7 ± 0.11% ID/g at 0.5 h to 22 ± 1.4% ID/g at 3 h in normal mice, and from 3.5 ± 0.12% ID/g to 25 ± 1.37% ID/g in tumor-bearing mice. Furthermore, although radioactive decay occurs, the short imaging window (0.5–3 h) and the relatively long half-life of ^99m^Tc (~ 6 h) result in minimal impact on the observed biodistribution within this timeframe. Consequently, the increasing bladder activity reflects sustained renal excretion rather than anomalous retention or tracer redistribution [64]. Additionally, the urinary bladder contains alpha-1 adrenoceptors, a type of adrenergic receptor. Specifically, the bladder neck and the proximal urethra exhibit a high density of these receptors. The primary function of alpha-1 adrenoceptors in the lower urinary tract is to facilitate the contraction of smooth muscle [65]. In the bladder, their activation leads to an increase in outlet resistance, which is crucial for urinary continence (the ability to retain urine). Tamsulosin acts as an alpha-blocker for these receptors, localizing to a high concentration in the urinary bladder, which causes the smooth muscles of the bladder to relax, thereby reducing bladder outlet resistance and improving urinary flow. Consequently, it is also used in the treatment of lower urinary tract symptoms associated with BPH. Furthermore, the compound showed minimal accumulation in the brain, consistent with its lipophilic nature [66]. The ability of the tracer to cross the blood-brain barrier may be attributed to Tamsulosin’s known affinity for alpha-1 adrenoceptors, particularly the α1A and α1D subtypes, which are expressed in the central nervous system [67]. This suggests that even limited penetration into the brain may yield specific pharmacological effects in targeted neural regions.

The pronounced tumor localization of [⁹⁹ᵐTc]Tc-Tamsulosin in Ehrlich carcinoma models can be attributed to a multifaceted mechanism involving physicochemical properties, molecular interactions, and tumor-specific pathophysiology. The compound’s moderate lipophilicity (Log *P* = 1.85 ± 0.03) enhances membrane permeability and passive diffusion, thereby contributing to increased intratumoral accumulation and a high target-to-non-target ratio (T/NT = 5.3 at 2 h p.i.). This selective uptake in the target tumor tissues (5.2 ± 0.02% ID/g) significantly surpasses that of adjacent non-target normal tissues (0.98 ± 0.01% ID/g), indicating the compound’s affinity for neoplastic environments. The structural inclusion of a sulfonamide moiety enhances binding specificity to malignant cells, while the acidic tumor microenvironment (pH 6.5–6.8) promotes protonation of amine groups, intensifying electrostatic interactions and receptor affinity [68]. This pH-dependent ion trapping further facilitates lysosomal sequestration within acidic intracellular compartments. Moreover, enhanced permeability and retention (EPR) effect, characterized by aberrant vasculature and impaired lymphatic drainage, enables passive accumulation of the radiotracer. Active targeting mechanisms also play a crucial role, with receptor-mediated endocytosis and transporter-assisted uptake supported by the expression of α₁A and α₁D adrenergic receptors on tumor cells and neovasculature [69]. Tamsulosin’s antagonism of these receptors may modulate local hemodynamics, reducing nutrient flow and enhancing tracer retention. The integration of these passive and active processes allows for deep tissue penetration and sustained retention, with their relative contributions influenced by tumor heterogeneity, including variations in vascular density, hypoxia, and receptor expression [70].

In contrast, normal skeletal muscle lacks the pathological features necessary for radiotracer accumulation, such as disrupted vasculature, resulting in minimal nonspecific uptake. These findings underscore the importance of tumor-selective biochemical and physiological alterations in optimizing radiopharmaceutical targeting. As illustrated in Fig. 8, the [⁹⁹ᵐTc]Tc-Tamsulosin complex demonstrates a superior tumor-to-non-target (T/NT) ratio of 5.3 at 2 h post-injection, exceeding several established radiotracers employed in solid tumor imaging, such as [⁹⁹ᵐTc]oseltamivir as (T/NT ratio of 4.55 at 3 h p.i), [^99m^Tc]Ifosfamide (T/NT ratio of 4.98 at 1 h p.i), [^99m^Tc]amitrole (T/NT ratio of 4.9 at 1 h p.i), [^99m^Tc]Mebeverine (T/NT ratio of 3.14 at 3 h p.i), and [^99m^Tc]gemcitabine (T/NT ratio of 4.9 at 2 h p.i) [17–21]. This comparative advantage highlights the compound’s ability to achieve pronounced tumor delineation with minimal background interference. The combination of high intratumoral retention and rapid systemic clearance positions [⁹⁹ᵐTc]Tc-Tamsulosin as a promising candidate for molecular imaging of cancer, offering enhanced diagnostic precision. Furthermore, its selective biodistribution profile supports potential utility in theranostic applications, where targeted radiotherapy may benefit from the same mechanisms driving its imaging efficacy.

## Conclusion

This study successfully developed and characterized the [⁹⁹ᵐTc]Tc-Tamsulosin complex as a promising radiopharmaceutical for tumor imaging. The tracer demonstrated high radiochemical yield (97.5 ± 0.12%), robust in vitro stability, and favorable lipophilicity, supporting its suitability for biological applications. In vivo biodistribution studies revealed selective and sustained uptake in Ehrlich solid tumors, with a peak tumor-to-non-target (T/NT) ratio of 5.3 at 2 h post-injection, surpassing several previously reported radiotracers. These findings support further investigation into the [⁹⁹ᵐTc]Tc-Tamsulosin complex as a radiopharmaceutical with dual utility in both imaging and therapeutic contexts.

## Data Availability

All experimental data were obtained at the Egyptian Atomic Energy Authority and subsequently incorporated into the manuscript.

## Declarations

### Preprint Sharing

This manuscript has not been previously published as a preprint.

## References

[1] Etzioni R, Urban N, Ramsey S, McIntosh M, Schwartz S, Reid B, et al. The case for early detection. Nat Rev Cancer. 2003;3:243–52. 12671663 10.1038/nrc1041

[2] Hassan RM, Abd-Allah WH, Salman AM, El-Azzouny AAS, Aboul-Enein MN. Design, synthesis and anticancer evaluation of novel 1,3-benzodioxoles and 1,4-benzodioxines. Eur J Pharm Sci. 2016;139:105045. 10.1016/j.ejps.2019.105045 31421253

[3] Gabriel JA, editor. The biology of cancer. Hoboken (NJ): Wiley; 2007.

[4] Ozaslan M, Karagoz I, Kilic I, et al. Ehrlich Ascites carcinoma. Afr J Biotechnol. 2011;10:2375–8.

[5] Nascimento FR, Cruz GV, Pereira PV, et al. Ascitic and solid Ehrlich tumor inhibition by *Chenopodium ambrosioides* L. treatment. Life Sci. 2006;78:2650–3. 16307762 10.1016/j.lfs.2005.10.006

[6] Fernandes PD, Guerra FS, Sales NM, et al. Characterization of the inflammatory response during Ehrlich ascitic tumor development. J Pharmacol Toxicol Methods. 2015;71:83–9. 25199596 10.1016/j.vascn.2014.09.001

[7] Pessina A, Brambilla P, Mocarelli P. Surface antigen on Ehrlich Ascites tumor cells. Biomedicine. 1980;33:105–9. 7000194

[8] Akinaga J, García-Sáinz JA, Pupo A. Updates in the function and regulation of α1-adrenoceptors. Br J Pharmacol. 2019;176(14):2343–57. 10.1111/bph.14617. 30740663 10.1111/bph.14617 PMC6592863

[9] Rattigan S, Appleby GJ, Edwards SJ, McKinstry WJ, Colquhoun EQ, Clark MG, et al. Alpha-adrenergic receptors in rat skeletal muscle. Biochem Biophys Res Commun. 1986;136(3):1071–7. 3013164 10.1016/0006-291x(86)90442-0

[10] Jarajapu YPR, McGrath JC, Hillier C, MacDonald A. The α1-adrenoceptor profile in human skeletal muscle resistance arteries in critical limb ischaemia. Cardiovasc Res. 2003;57(2):554–62. 12566128 10.1016/s0008-6363(02)00669-7

[11] Mishra RC, Rahman MM, Davis MJ, Wulff H, Hill MA, Braun AP. Alpha1-adrenergic stimulation selectively enhances endothelium-mediated vasodilation in rat cremaster arteries. Physiol Rep. 2018;6(9):e13703. 10.14814/phy2.13703. 29756401 10.14814/phy2.13703 PMC5949301

[12] Abrams P, Schulman CC, Vaage S. Tamsulosin, a selective α1c-adrenoceptor antagonist: a randomized, controlled trial in patients with benign prostatic obstruction. Br J Urol. 1995;76:325–36. 7551841 10.1111/j.1464-410x.1995.tb07709.x

[13] Schulman CC. Tamsulosin, the first prostate-selective α1A-adrenoceptor antagonist: analysis of a multinational, multicentre, open-label study assessing the long-term efficacy and safety in patients with benign prostatic obstruction. Eur Urol. 1996;29:145–54. 8647140

[14] Shams El-Din HA, Zaki EG. Synthesis, 99mTc-radiolabeling and in vivo evaluation of a new sulphonamide derivative for solid tumor imaging. J Radioanal Nucl Chem. 2020;326(1):129–36.

[15] Ghorab MM, Ceruso M, Alsaid MS, Nissan YM, Arafa RK, Supuran CT. Novel sulfonamides bearing pyrrole and pyrrolopyrimidine moieties as carbonic anhydrase inhibitors: synthesis, cytotoxic activity and molecular modeling. Eur J Med Chem. 2014;87:186–96. 10.1016/j.ejmech.2014.09.059. 25255434 10.1016/j.ejmech.2014.09.059

[16] Hendee WR, Ritenour ER. Medical imaging physics. Hoboken (NJ): Wiley; 2003.

[17] Challan SB, Khater SI, Rashad AM. Preparation, molecular modeling and in-vivo evaluation of ^99m^Tc-Oseltamivir as a tumor diagnostic agent. Int J Radiat Res. 2022;20:635–42.

[18] Motaleb MA, Selim AA, El-Tawoosy M, Sanad MH, El-Hashash MA. Synthesis, radiolabeling and biological distribution of a new dioxime derivative as a potential tumor imaging agent. J Radioanal Nucl Chem. 2017;314(3):1517–22.

[19] Motaleb MA, El-Safoury DM, Abd-Alla WH, Awad GA, Sakr TM. Radiosynthesis, molecular modeling studies and biological evaluation of ^99m^Tc-Ifosfamide complex as a novel probe for solid tumor imaging. Int J Radiat Biol. 2018;94(12):1134–41. 30373490 10.1080/09553002.2019.1524945

[20] Essa BM, Sakr TM, Khedr MA, El-Essawy FA, El-Mohty AA. ^99m^Tc-amitrole as a novel selective imaging probe for solid tumor: in silico and preclinical pharmacological study. Eur J Pharm Sci. 2015;76:102–9. 25956074 10.1016/j.ejps.2015.05.002

[21] Essam HM, Refaye MS, El-Sharawy DM. Radiolabelling and biological assessment of ^99m^Tc-Mebeverine as a possible tracer for solid tumor diagnosis. Egypt J Radiat Sci Appl. 2021;34(1):21–6.

[22] Ibrahim AB, Sakr TM, Khoweysa OMA, Motaleb MA, Abd El-Bary A, El-Kolaly MT. Formulation and preclinical evaluation of ^99m^Tc-gemcitabine as a novel radiopharmaceutical for solid tumor imaging. J Radioanal Nucl Chem. 2014;302:179–86.

[23] El-Sharawy DM, Khater SI, Sherif NH, Hassan HM, Elmaidomy AH. ^99m^Tc-Luteolin: radiolabeling, in silico ADMET and biological evaluation as a natural tracer for tumor imaging. J Radiat Res Appl Sci. 2021;14(1):125–32.

[24] Lin J, Qiu L, Lv G, Li K, Wang W, Liu G, et al. Synthesis and preliminary biological evaluation of a <Superscript>99m</Superscript>Tc-chlorambucil derivative as a potential tumor imaging agent. J Label Compd Radiopharm. 2017;60:116–23. 10.1002/jlcr.3481 27862213

[25] Sakr TM, El-Safoury DM, Awad GA, Motaleb MA. Biodistribution of ^99m^Tc-sunitinib as a potential radiotracer for tumor hypoxia imaging. J Label Compd Radiopharm. 2013;56:392–5. 10.1002/jlcr.3060 24285479

[26] Sanad MH, Marzook F, Saleh GM, Farag AB, Talaat HM. Radiolabeling, preparation, and bioevaluation of ^99m^Tc-Azathioprine as a potential targeting agent for solid tumor imaging. Radiochemistry. 2019;61:478–82.

[27] Ibrahim IT, Attallah KM. Synthesis of 99 m Tc-L-carnitine as a model for tumor imaging. Radiochemistry. 2021;54(4):407–11.

[28] Motaleb MA, Selim AA, El-Tawoosy M, Sanad MH, El-Hashash MA. Synthesis, characterization, radiolabeling and biodistribution of a novel cyclohexane dioxime derivative as a potential candidate for tumor imaging. Int J Radiat Biol. 2018;94(6):590–6. 29659318 10.1080/09553002.2018.1466067

[29] Sanad MH, Borai EH. Performance characteristics of biodistribution of ^99m^Tc-cefprozil for in vivo infection imaging. J Anal Sci Technol. 2014;5(1):32.

[30] Sanad MH, Ibrahim AA, Talaat HM. Synthesis, bioevaluation and gamma scintigraphy of ^99m^Tc-N-2-(Furylmethyl iminodiacetic acid) complex as a new renal radiopharmaceutical. J Radioanal Nucl Chem. 2018;315(1):57–63.

[31] El-Kawy OA, Sanad MH, Marzook F. ^99m^Tc-Mesalamine as potential agent for diagnosis and monitoring of ulcerative colitis: labelling, characterisation and biological evaluation. J Radioanal Nucl Chem. 2016;308(1):279–86.

[32] Sanad MH, Challan SB, Essam HM, Abdou FY, Farag AB. Design of a novel complex ^99m^Tc-Nilutamide as a tracer for prostate cancer disorder detection in mice. Radiochim Acta. 2025;113(3):213–28.

[33] Sanad MH, Borai EH, Fouzy ASM. Chromatographic separation and utilization of labeled ^99m^Tc-valsartan for cardiac imaging. J Mol Imag Dyn. 2014;4(114):2.

[34] Massoud A, Challan SB, Maziad N. Characterization of polyvinyl pyrrolidone (PVP) with technetium-99m and its accumulation in mice. J Macromol Sci Pure Appl Chem. 2021;58:408–18.

[35] Sanad MH, Challan SB, Essam HM, Massoud A. Assessment of radiolabeled L-Carnitine for hepatotoxicity imaging in rats. Radiochemistry. 2023;65:101–13.

[36] Challan SB, Massoud A. Radiolabeling of graphene oxide by Technetium-99m for infection imaging in rats. J Radioanal Nucl Chem. 2017;314(3):2189–99.

[37] Sanad MH, Eyssa HM, Challan SB, Farag AB, Abdou FY, Soliman AM, et al. Facile one-pot strategy for brain imaging using radiolabeled [^99m^Tc]-tricarbonyl Histamine complex in mice. Egypt J Chem. 2024;68:37–45.

[38] Sanad MH, Marzook EA, Challan SB. Radioiodination of Olmesartan Medoxomil and biological evaluation of the product as a tracer for cardiac imaging. Radiochim Acta. 2018;106:329–36.

[39] Moustapha ME, Motaleb MA, Sanad MH. Synthesis and biological evaluation of ^99m^Tc-labetalol for β1-adrenoceptor-mediated cardiac imaging. J Radioanal Nucl Chem. 2016;309(2):511–6.

[40] Thimmaraju MK, Rao V, Kumar PS. RP HPLC method for the determination of Tamsulosin in bulk and pharmaceutical formulations. J Appl Pharm Sci. 2011;177 – 80.

[41] Challan SB, Abd El-Kareem MSM, Rashad AM, Khater SI. Characterization and bioevaluation of radioiodinated oseltamivir phosphate for inflammation imaging as a new perspective. Bioorg Chem. 2025;165:1–13. 10.1016/j.bioorg.2025.109050. 10.1016/j.bioorg.2025.109050 41032955

[42] Sanad MH, Marzook FAM, Challan SB, Essam HM, Farag AB. Radioiodination and biological assessment of olsalazine as a highly selective radiotracer for ulcerative colitis imaging in mice. Arab J Nucl Sci Appl. 2023;56(3):105–20.

[43] Sanad MH, Challan SB, Abdou FY, El-Desawy M, Essam HM. Radioiodination of silodosin with ^131^I as a selective drug for prostate imaging in mice. Cancer Biother Radiopharm. 2025;40(6):1–16. 10.1089/cbr.2025.0015. 40340570 10.1089/cbr.2025.0015

[44] Kowalsky RJ. Technetium radiopharmaceutical chemistry. Albuquerque (NM). University of New Mexico Health Sciences Center College of Pharmacy. 2006.

[45] Costa B, Ilem-Özdemir D, Santos-Oliveira R. Technetium-99m metastable radiochemistry for pharmaceutical applications: old chemistry for new products. J Coord Chem. 2019;72:1759–78.

[46] Wilde MI, McTavish D. Tamsulosin: a review of its pharmacological properties and therapeutic potential in the management of symptomatic benign prostatic hyperplasia. Drugs. 1996;52(6):883–98. 8957159 10.2165/00003495-199652060-00012

[47] Green CH. Technetium-99m production issues in the united Kingdom. J Med Phys. 2012;37(2):66–71. 22557795 10.4103/0971-6203.94740 PMC3339145

[48] Sanad MH, Fouzy ASM, Sobhy HM, Hathout AS, Hussain OA. Tracing the protective activity of *Lactobacillus plantarum* using technetium-99m-labeled zearalenone for organ toxicity. Int J Radiat Biol. 2018;94(12):1151–8. 30273080 10.1080/09553002.2019.1524990

[49] Motaleb MA, Sanad MH, Selim AA, El-Tawoosy M, Abd-Allah M. Synthesis, characterization, and radiolabeling of heterocyclic bisphosphonate derivative as a potential agent for bone imaging. Radiochemistry. 2018;60(2):201–7.

[50] Sanad MH, Marzook EA, El-Kawy OA. Radiochemical and biological characterization of ^99m^Tc-oxiracetam as a model for brain imaging. Radiochemistry. 2017;59(6):624–9.

[51] Motaleb MA, El-Tawoosy M, Mohamed SB, Borei IH, Ghanem HM, Massoud AA. ^99m^Tc-labeled teicoplanin and its biological evaluation in experimental animals for detection of bacterial infection. Radiochem. 2014;56:544–9.

[52] Bekheet S, El-Tawoosy M, Massoud A, Borei IH, Ghanem HM, Motaleb MA. ^99m^Tc-labeled Ceftazidime and biological evaluation in experimental animals for detection of bacterial infection. Am J Biochem. 2014;4:15.

[53] Challan SB, Massoud AA, El-Tawoosy M, Motaleb MA, Borei IH, Ghanem HM. ^99m^Tc-labeled erythrocin and biological evaluation in mice for detection of bacterial infection. Asian J Phys Chem Sci. 2018;5:1–13.

[54] Geskovski N, Kuzmanovska S, Crcarevska MS, et al. Comparative biodistribution studies of technetium-99m radiolabeled amphiphilic nanoparticles using three different reducing agents during the labeling procedure. J Labelled Comp Radiopharm. 2013;56:689–95. 24339006 10.1002/jlcr.3097

[55] Safaa B, Mohamed. Radiolabeling of some antimicrobial compounds for inflammation imaging and its biological evaluation in mice, PhD thesis. Cairo (Egypt): Ain Shams University, Faculty of Science, Biochemistry Department. 2015.

[56] Marzook EA, Talaat HM, Challan SB. Comparative biological evaluation of ^99m^Tc-timonacic acid prepared using different reducing agents as a complex for hepatobiliary imaging. Radiochemistry. 2018;60(3):309–15.

[57] Sanad MH, Eyssa HM, Marzook FA, Farag AB, Elrefaei A, Fouzy ASM, et al. Radiocomplexation, biological evaluation, and characterization of [^99m^Tc]-5-[(3-carboxy-4-hydroxyphenyl) diazenyl]-2-hydroxybenzoic acid as a novel agent for imaging of ulcerative colitis in mice. Radiochemistry. 2023;65:378–86.

[58] Challan SB, Marzook FA, Massoud A. Synthesis of radioiodinated carnosine for hepatotoxicity imaging induced by carbon tetrachloride and its biological assessment in rats. Radiochim Acta. 2020;108(5):397–408.

[59] Sanad MH, Challan SB. Radioiodination and biological evaluation of Rabeprazole as a peptic ulcer localization radiotracer. Radiochem. 2017;59:307–12.

[60] Sanad MH, Challan SB, Marzook FA, Abd-Elhaliem SM, Marzook EA. Radioiodination and biological evaluation of cimetidine as a new highly selective Radiotracer for peptic ulcer disorder detection. Radiochim Acta. 2021;109.

[61] Challan SB, El-Ghany EA, Amin AM, Hanafi HA, Borai IH, Moselhy SS. Radio-iodination of oxytocin as an imaging agent for oxytocin receptors in mice. Proceedings of the 16th Arab Conference on the Peaceful Uses of Atomic Energy; 2024; Dead Sea, Hashemite Kingdom of Jordan.

[62] Amin AM, Mohamed SB, Moselhy SS, Boraei IH. Technetium-99m salbutamol: a single photon emission imaging agent for β2-adrenoceptor and regional cerebral blood flow. Arab J Nucl Sci Appl. 2009;42(2):34–41.

[63] Gill SS, Bai A. Beta testing the potential link between the alpha antagonist tamsulosin and dementia. Pharmacoepidemiol Drug Saf. 2018;27(3):349–50. 29341324 10.1002/pds.4382

[64] Madersbacher S, Michel MC. Tamsulosin and the risk of dementia in older men with benign prostate hyperplasia. Eur Urol. 2018;74(4):522–3. 30057132 10.1016/j.eururo.2018.07.013

[65] Kosilov K, Kuzina I, Kuznetsov V, Gainullina Y, Kosilova L, et al. Cognitive functions and health-related quality of life in men with benign prostate hyperplasia and symptoms of overactive bladder when treated with a combination of tamsulosin and solifenacin in a higher dosage. Aging Male. 2018;21(2):121–9. 29113548 10.1080/13685538.2017.1398723

[66] Michel MC, Goepel M. Differential α1-adrenoceptor labeling by [3H] Prazosin and [3H] Tamsulosin. Eur J Pharmacol. 1998;342(1):85–92. 9544796 10.1016/s0014-2999(97)01419-2

[67] Kato Y, Ozawa S, Miyamoto C, Maehata Y, Suzuki A, Maeda T, et al. Acidic extracellular microenvironment and cancer. Cancer Cell Int. 2013;13:89. 10.1186/1475-2867-13-89. 24004445 10.1186/1475-2867-13-89 PMC3849184

[68] Kurimoto S, Moriyama N, Hamada K, et al. Quantitative autoradiography of α1-adrenoceptors with [3H]tamsulosin in human hypertrophied prostate using computerized image analysis. Histochem J. 1995;27:1007–13. 10.1007/BF02389691. 8789402

[69] Wang X, et al. Current status and future prospects of molecular imaging in targeting the tumor immune microenvironment. Front Immunol. 2025;16:1518555. 39911388 10.3389/fimmu.2025.1518555 PMC11794535

[70] Salavati H, Debbaut C, Pullens P, Ceelen W. Interstitial fluid pressure as an emerging biomarker in solid tumors. Biochim Biophys Acta Rev Cancer. 2022;1877(5):188792. 10.1016/j.bbcan.2022.188792. 36084861 10.1016/j.bbcan.2022.188792

